# Transport proteins of parasitic protists and their role in nutrient salvage

**DOI:** 10.3389/fpls.2014.00153

**Published:** 2014-04-29

**Authors:** Paul Dean, Peter Major, Sirintra Nakjang, Robert P. Hirt, T. Martin Embley

**Affiliations:** The Medical School, Institute for Cell and Molecular Biosciences, Newcastle UniversityNewcastle upon Tyne, UK

**Keywords:** transporter, transport, parasite, protist, protozoa, hexose, purine, amino acid

## Abstract

The loss of key biosynthetic pathways is a common feature of important parasitic protists, making them heavily dependent on scavenging nutrients from their hosts. This is often mediated by specialized transporter proteins that ensure the nutritional requirements of the parasite are met. Over the past decade, the completion of several parasite genome projects has facilitated the identification of parasite transporter proteins. This has been complemented by functional characterization of individual transporters along with investigations into their importance for parasite survival. In this review, we summarize the current knowledge on transporters from parasitic protists and highlight commonalities and differences in the transporter repertoires of different parasitic species, with particular focus on characterized transporters that act at the host-pathogen interface.

## Introduction

Central to a parasitic lifestyle is the need to acquire nutrients from the host as parasites have often lost the ability to synthesize key nutrients *de novo*. Salvage of nutrients by parasitic protists is made possible by plasma membrane transporter proteins that represent potential therapeutic targets and therefore identifying them and understanding their function is important. Our knowledge on the biology and pathogenesis of parasitic protists comes from relatively few model species that have been studied in detail, including the kinetoplastids *Trypanosoma* and *Leishmania*; the apicomplexans *Plasmodium, Toxoplasma* and *Cryptosporidium*; the microsporidians *Trachipleistophora hominis* and *Encephalitozoon cuniculi*; the excavate *Trichomonas vaginalis* and the amoeba *Entamoeba histolytica* (the biology of these parasites have been reviewed previously Clarke et al., 2010; Sibley, 2011). Progress in understanding the transport systems of these parasites has been aided by the availability of their genome sequences, facilitating the identification of transporter repertoires, and functional characterization of individual transporters.

**Figure 1:**
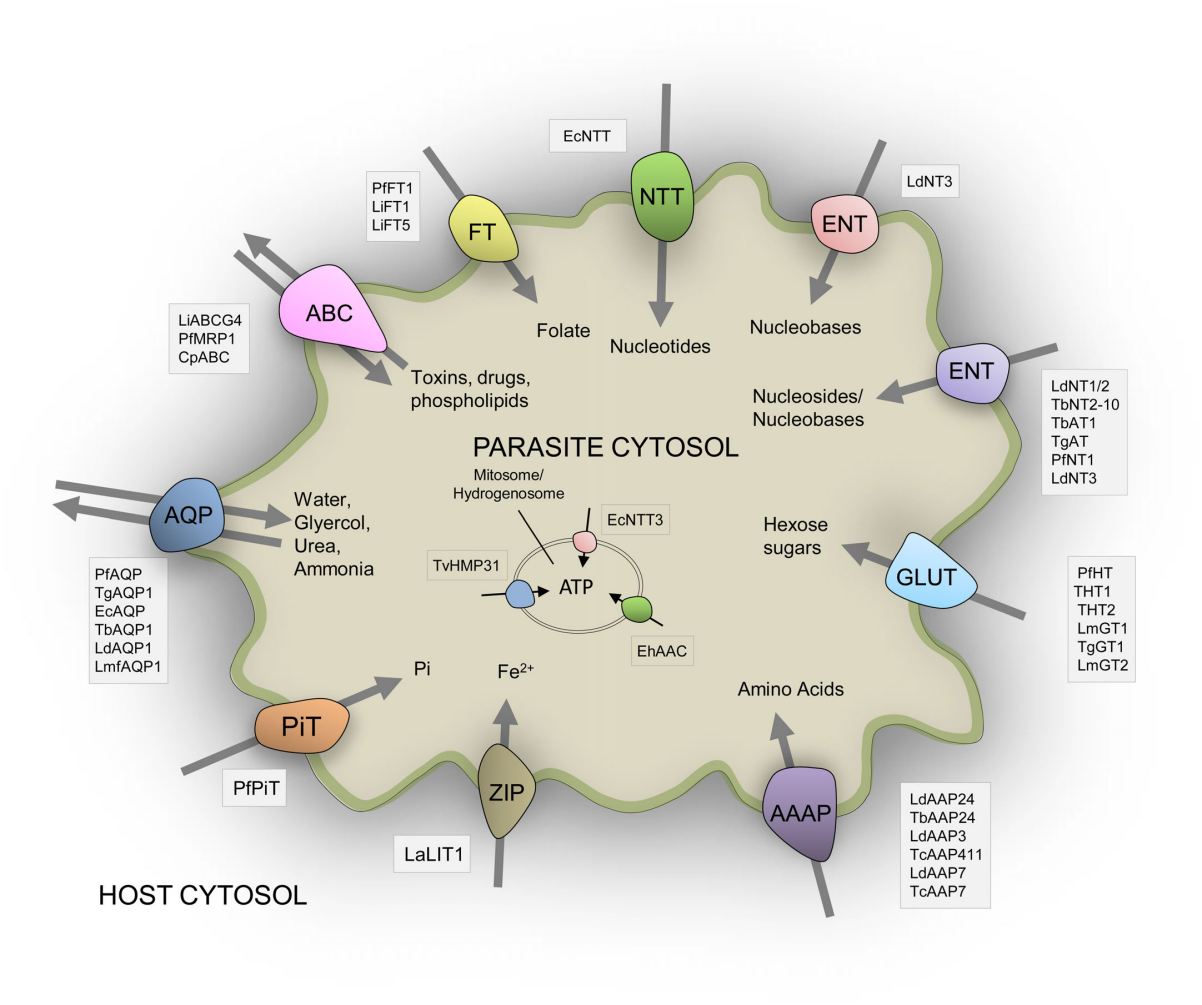
**Schematic representation of the diversity of experimentally-verified transport proteins located on the surface membrane of parasitic protists based upon published data**. All transporters shown are given and discussed in the main text. Three transporters located in mitochondria derived organelles are also indicated. See main text for all abbreviations used for species and transporters.

**Figure 2:**
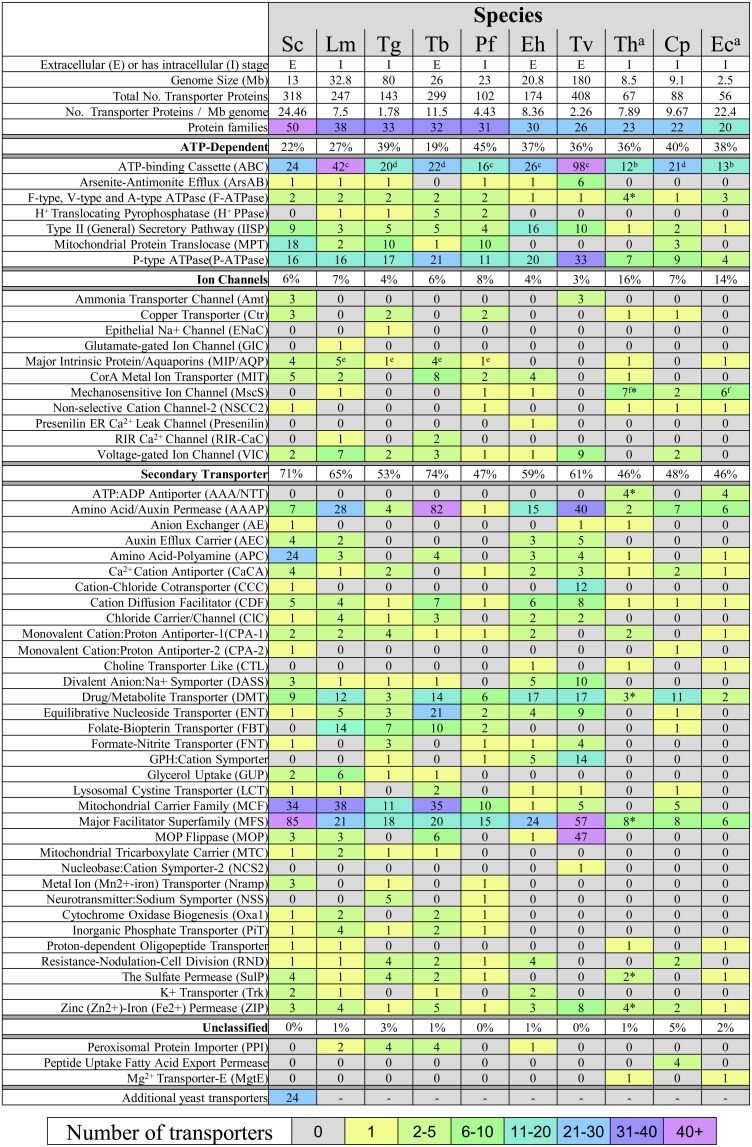
**Transport protein repertoires of parasitic protists**. Prediction of transporters was taken from the literature where indicated or obtained from the TransportDB database (Ren et al., 2007). Nomenclature of transporters is consistent with that given in TransportDB. Transporter proteins shown are those only found in the selected parasites, as those having no hits in any parasite were omitted. Most transporters are not experimentally verified and their subcellular location has not been determined (see Table 1). Key: ^*^Expanded protein families from the analysis of 9 species of microsporidia. The 24 additional yeast transporters can be found in the TransportDB database and comprise 10 transporter families. (a) Data for microsporidian transporters were taken from (Heinz et al., 2012). (b) Includes proteins that possess an ABC transporter domain (PFAM:PF00005, *e*-value cutoff < 1e-5) using InterProScan. 4.8. Values taken from (c) (Kay et al., 2012), (d) (Sauvage et al., 2009), (e) (Beitz, 2005), and (f) (Heinz et al., 2012). Cp, *Cryptosporidium parvum*; Eh, *Entamoeba histolytica*; Lm, *Leishmania major*; Pf, *Plasmodium falciparum*; Tg, *Toxoplasma gondii*; Tv, *Trichomonas vaginalis*; Tb, *Trypanosoma brucei*; Th, *Trachipleistophora hominis*; Ec, *Encephalitozoon cuniculi*. *Sc, Saccharomyces cerevisiae*.

The number of recognized transporter families in selected parasitic protists (20–38 families; Figure 2) is markedly reduced compared to free-living microorganisms such as yeast (50 families; Figure 2; Gardner et al., 2002; Carlton et al., 2007; Ren et al., 2007), possibly reflecting the diverse range of niches encountered by the latter. Strict intracellular parasites such as microsporidians, possess a plasma membrane that is only exposed when inside a host cell, and this specific niche correlates with the low number of transporter families in these parasites (Figure 2). Parasites in general also face specific challenges that favor a minimalistic lifestyle including the need to reduce antigenicity of their exposed surfaces within the host and the need to multiply rapidly upon infection. An overview of the literature suggests that the reduction in parasite transporter proteins is likely balanced by increased functional diversification of these transporters including a broadening of their substrate range and alterations in transport mechanism. In this review, we mainly focus on parasite transporters acting at the parasite's plasma membrane that are responsible for nutrient salvage from the host. An overview of the transporters discussed is given in Figure 1 and Table 1.

**Table 1:**
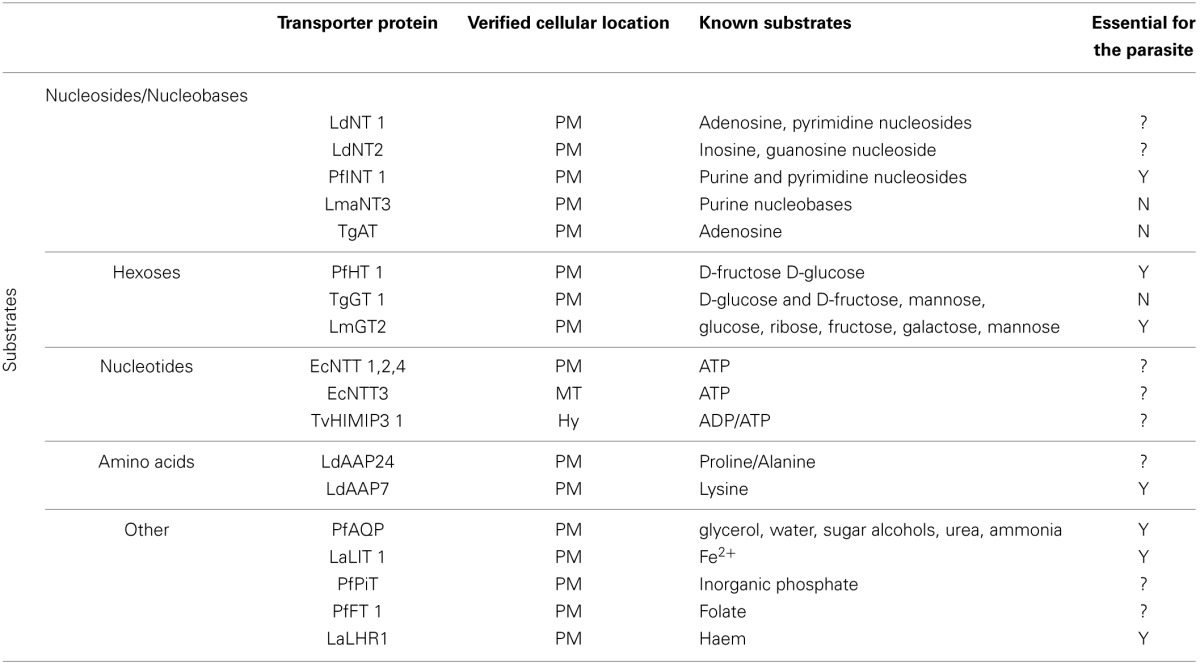
**Features of experimentally-verified parasite transport proteins**.

*An overview of the features of characterized parasite transporters. In all cases, subcellular location was determined experimentally as described in the text. A question mark indicates either that the essentiality of the transporter was not determined or this information could not be found in the literature. Details of individual transporters are discussed in the main text*.

## Purine and pyrimidine transporters

The need for parasites to replicate rapidly during host colonization places a high demand on DNA and RNA synthesis and requires purine and pyrimidine nucleotides. In addition, nucleotides are essential sources of chemical energy, and act as co-factors and precursors in a wide variety of metabolic reactions. Parasitic protists typically lack the early ATP-dependent steps in the *de novo* biosynthesis of purines (and sometimes pyrimidines) (Carter et al., 2001) and hence they need to import the missing substrates using dedicated transporters. Although the most common sources of host-derived purines and pyrimidines are nucleosides and nucleobases (see below), some parasites including microsporidia import nucleotides directly (Tsaousis et al., 2008). Purine and pyrimidine transport in parasitic protists is well established in the literature (Landfear et al., 2004; De Koning et al., 2005) but only relatively recently have the transporters been investigated in detail at the molecular level and their importance for the parasites investigated.

### Nucleoside and nucleobase ENT transporters

The nucleoside and nucleobase transporters that are characterized in parasitic protists all belong to the equilibrative nucleoside transporter (ENT) family, which have eleven transmembrane domains and are widely found in animals and plants (Acimovic and Coe, 2002) (although homology to bacterial transporters has been postulated Acimovic and Coe, 2002). Typical ENT transporters are so named because they equilibrate nucleoside and nucleobase concentration down a concentration gradient rather than against it (Acimovic and Coe, 2002). Most genetic and molecular studies on parasite ENT transporters have been done on the kinetoplastids *Leishmania* and *Trypanosoma*, with some related studies on *Plasmodium* and *Toxoplasma* (for review see Landfear et al., 2004). *Leishmania donovani* has two distinct and high-affinity nucleoside transporters called LdNT1 and LdNT2 with non-overlapping substrate specificities [LdNT1 transports adenosine and pyrimidine nucleosides (Vasudevan et al., 1998); LdNT2 transports inosine and guanosine nucleosides (Carter et al., 2000b)]. A third ENT transporter, LmaNT3 in *Leishmania major* (an ortholog of *L. donovani* LdNT3) was found to have high affinity for purine nucleobases (adenine and guanine) but not for pyrimidine nucleobases or nucleosides (Sanchez et al., 2004). All three transporters have been localized to the parasite plasma membrane and have been functionally characterized (Vasudevan et al., 2001; Arastu-Kapur et al., 2003; Galazka et al., 2006). LdNT1 and 2 display a 100-fold increase in substrate affinity compared with mammalian ENT transporters (Landfear et al., 2004), thus enabling them to salvage nutrients effectively from the host (Landfear et al., 2004). Heterologous expression in *Xenopus* oocytes showed that LdNT1/2 are electrogenic proton symporters (Stein et al., 2003), unlike mammalian ENT transporters, that presumably use an electrochemical gradient at the parasite plasma membrane to effect transport (Stein et al., 2003). Thus, although typical ENT family members facilitate the diffusion of substrates down concentration gradients, the *Leishmania* transporters may be concentrative rather than equilibrative transporters (Stein et al., 2003). It would be interesting to test whether this transport mechanism is a general adaptation by parasite ENT transporters, increasing the efficiency of nutrient salvage from the host.

Mutational analysis of the *Leishmania* ENT transporters has revealed important insights into the amino acid residues affecting substrate specificity. A single amino acid substitution (G183D) in a transmembrane domain of LdNT1 decreased its transport capacity for adenosine—a change that was responsible for the organism's resistance to the adenosine analog tubercidin (Vasudevan et al., 2001). By contrast, a different substitution—G183A—impaired the transport of pyrimidines, thus elegantly demonstrating that changing a single residue can selectively influence substrate specificity (Vasudevan et al., 2001). It was found that the G183 residue was located on the hydrophilic face of the transmembrane domain involved in nucleoside translocation, and was subsequently shown to be essential for transporter function (Valdes et al., 2004)—a finding also observed with human ENTs (SenGupta et al., 2002). In a separate study, a conservative residue change (K153R) in LdNT1, conferred an ability to transport inosine—a new substrate for this transporter, thus extending its substrate range (Valdes et al., 2006). Mutational analysis of LdNT2 revealed that a single residue change can dramatically affect the affinity of the transporter for its substrate, as a N175I substitution caused a 10-fold increase in the apparent K_m_ for inosine (Arastu-Kapur et al., 2005). These data demonstrate very clearly that changes at the level of single amino acids can have dramatic effects on the substrate range and affinities of *Leishmania* ENT transporters.

ENT transporters identified in other parasites include several in *Trypanosoma brucei* (termed TbNT2-10) which transport purine nucleosides and nucleobases (Sanchez et al., 2002; Landfear et al., 2004), a single *T. brucei* transporter (TbAT1) that transports adenine and adenosine (Maser et al., 1999), and low-affinity adenosine ENT transporters in *Toxoplasma gondii* (TgAT) and *Plasmodium falciparum* (PfNT1) (Chiang et al., 1999; Carter et al., 2000a)—the latter being localized to the parasite plasma membrane (Rager et al., 2001). PfNT1 has been reported to transport both purines and pyrimidine nucleosides (Carter et al., 2000a) while TgAT1 has a low affinity for adenosine. Genetic disruption of the PfNT1 gene revealed that it is essential for purine nucleoside uptake and for parasite survival (El Bissati et al., 2006) while knockouts of TgAT1 suggest it is a non-essential gene (Chiang et al., 1999). However, nucleoside import studies suggested *T. gondii* possesses a second unknown nucleoside transporter with much higher substrate affinities (De Koning et al., 2003). More recent work has revealed that in addition to PfNT1, a further three ENT transporters are present in the genome of *P. falciparum* (named PfNT2-4; Martin et al., 2005) with functional characterization revealing that PfNT4 transports adenine nucleobases and nucleosides (Frame et al., 2012) while PfNT2 may transport uridine (Downie et al., 2010) but was localized to the parasite endoplasmic reticulum (Downie et al., 2010)—clearly different to most other characterized ENTs, which are typically found in the plasma membrane (Table 1; Rager et al., 2001; Landfear et al., 2004). Taken together, the functional data on the ENT transport family in parasitic protists suggests these transporters enable the parasites to salvage a broad range of nucleosides and nucleobases from the host, which can then be converted into nucleotides within the parasite.

### The NTT family of nucleotide transporters

Microsporidian genome sequences reveal they do not possess ENT-type nucleoside transporters but instead have a dedicated family of transport proteins that have been shown to transport nucleotides including ATP (Tsaousis et al., 2008; Heinz et al., 2012). Microsporidians have undergone massive gene loss in the transition to obligate intracellular parasitism including genes for the early steps of purine and pyrimidine biosynthesis needed to make DNA and RNA, and for ATP production by oxidative phosphorylation (Nakjang et al., 2013). Energy generation in spores appears to depend on a glycolytic pathway that is down-regulated in replicating intracellular parasites (Heinz et al., 2012) that must therefore import ATP from infected host cells (Tsaousis et al., 2008). Microsporidian nucleotide transport (NTT) proteins are thought to have been acquired by lateral gene transfer from intracellular bacterial pathogens (Greub and Raoult, 2003) such as *Chlamydia* or *Rickettsia*, that like microsporidians are unable to make nucleotides *de novo* (Horn et al., 2004). Recently, an NTT gene was also identified in the genome of the Cryptomycotan species *Rozella allomycis* (James et al., 2013), an obligate intracellular parasite that appears to be a sister group of the Microsporidia (James et al., 2013). This suggests that acquisition of bacterial NTT genes likely occurred in the common ancestor of Microsporidia and Cryptomycota—an event that may be key to the development of their shared obligate intracellular lifestyle.

The genome of the microsporidian *Encephalitozoon cuniculi* contains four NTTs (EcNTT1-4) and available genome sequences of all other microsporidia show one or more of these transporters (Corradi et al., 2009; Heinz et al., 2012)—for example *Spraguea lophii* has six putative NTTs (Campbell et al., 2013). All four EcNTTs transport ATP with high affinity when expressed in *E. coli* and competition assay data suggests that they may transport other nucleotides (Tsaousis et al., 2008). This is not surprising as bacterial NTTs transport a broad range of pyrimidine and purine nucleotides including NAD (Haferkamp et al., 2004; Audia and Winkler, 2006; Haferkamp et al., 2006; Knab et al., 2011). Three of the four EcNTTs are located in the parasite plasma membrane while one (EcNTT3) is localized to the mitosome, a remnant mitochondrial organelle (Tsaousis et al., 2008) that is not capable of generating its own ATP. Thus, EcNTT3 appears to provide the mitosome with ATP needed to support its metabolism (Goldberg et al., 2008; Tsaousis et al., 2008). Interestingly, liposomal transport assays with the *Protochlamydia amoebophila* transporter PamNTT1, revealed that the transport mechanism is independent of membrane potential (Trentmann et al., 2007). This is fundamentally different to other adenine nucleotide transporters such as the mitochondrial ADP/ATP carrier (AAC) family (see below) and may have physiological relevance, as mitosomes do not possess an electron transport chain and therefore may be unable to generate a membrane potential. Furthermore, the nature of any membrane potential at the microsporidian plasma membrane is unknown and may be too small for parasite ENT transporters (described above) to function, possibly explaining why NTTs and not ENT transporters are found in microsporidia (Ren et al., 2007) (Figure 2). As described above, ENT transporters at least for *Leishmania* seem to be active proton symporters (Stein et al., 2003)—utilizing electrochemical gradients to facilitate import (Landfear et al., 2004). Thus, although ENTs are typically found in other parasitic species, unlike the NTTs they may not be able to function effectively in microsporidia.

### Nucleotide transport by MCF proteins in mitochondria-related organelles

The mitochondrial carrier family (MCF) comprise structurally-related transport proteins responsible for translocating a broad range of solutes across the mitochondrial inner membrane—between the mitochondrial matrix and the cell cytosol (Palmieri et al., 2011). Free-living eukaryotes such as yeast have over thirty MCF proteins according to the TransportDB database (Ren et al., 2007) while MCF in parasitic protists are markedly reduced (Figure 2)—reflecting the decrease in metabolic capability of mitochondria in these species (Hjort et al., 2010). Indeed, some parasites possess highly reduced mitochondrial organelles called mitosomes or hydrogenosomes, that have lost many metabolic pathways except for iron-sulphur (FeS) cluster biosynthesis (Hjort et al., 2010). However, although FeS cluster formation requires a supply of several substrates including iron, cysteine, ATP and NADPH, the genomes of many parasitic protists have few, if any MCF proteins (Figure 2). For example, no MCF proteins are evident in the genomes of the microsporidians *E. cuniculi* (Katinka et al., 2001) or *T. hominis* (Heinz et al., 2012), raising the question of how substrate transport is achieved in their mitosomes. Here, we briefly discuss the few hydrogenosomal and mitosomal MCF transporters that have been characterized so far.

ADP/ATP carriers (AAC) are a sub-family of the MCF that mediate the exchange of mitochondrial-generated ATP with cytosolic ADP in model organisms such as yeast and humans (Kunji, 2004). In mitosomes and hydrogenosomes, the opposite is likely—ATP import in exchange for ADP export, suggesting that these parasite AAC transporters (see below) may be operating in reverse. An AAC-type transporter has been functionally characterized in *Neocallimastix patriciarum*, a non-parasitic, but hydrogenosome-containing anaerobic fungus (Voncken et al., 2002). This AAC was found in the hydrogenosome (van der Giezen et al., 2002; Voncken et al., 2002) and functional analysis showed that it mediated ADP/ATP exchange that was inhibited by compounds known to block classical mitochondrial-type AAC (van der Giezen et al., 2002). In addition, the *N. patriciarum* AAC complemented yeast mutants (van der Giezen et al., 2002), indicative of functional conservation of the transporter. By contrast, no AAC ortholog was found in the genome of the hydrogenosome-containing species *Trichomonas vaginalis* (Dyall et al., 2000; Tjaden et al., 2004), although five MCF proteins were found (Dyall et al., 2000; Tjaden et al., 2004). One of these MCF proteins, called HMP31, was highly abundant within the hydrogenosome-enriched fraction (Dyall et al., 2000). Functional characterization of the HMP31 ortholog from *Trichomonas gallinae* in *E. coli* demonstrated that it was able to transport ADP and ATP (Tjaden et al., 2004) but it was not inhibited by bongkrekic acid, a known inhibitor of classical AAC.

There is a debate about whether the mitosomes of the anaerobic amoeba *Entamoeba histolytica* participate in FeS cluster biosynthesis (Ali et al., 2004; Mi-ichi et al., 2009; Dolezal et al., 2010; Maralikova et al., 2010), but the organelle does have at least one important metabolic function for the parasite. Proteomic analysis of *E. histolytica* mitosomal-enriched fractions revealed the presence of proteins involved in sulphate activation, and a mitosomal location was confirmed by immunolocalization and biochemical analyses (Mi-ichi et al., 2009). The sulphate activation pathway requires substrates including ATP (Bradley et al., 2009) and two MCF transporters linked to this process have been found in the *E. histolytica* mitosome. The first was an ADP/ATP carrier that is phylogenetically distinct from archetypal AACs and found to be enriched in mitosome-containing fractions of *E. histolytica* (Chan et al., 2005). Functional characterization revealed the ability of this MCF to transport ATP and ADP (Chan et al., 2005). However, unlike classical mitochondrial AACs, which depend on electrogenic transport mechanisms to drive ADP/ATP translocation, the function of the *E. histolytica* AAC was not dependent on a membrane potential (Chan et al., 2005), possibly reflecting a lack of a membrane potential in the *Entamoeba* mitosome. The second *E. histolytica* MCF, termed EhPiC, was identified using a hidden Markov model based on defining features of the MCF family (Dolezal et al., 2010). EhPiC was functionally characterized as a phosphate transporter that was able to complement yeast mutants lacking a dominant phosphate transporter (Dolezal et al., 2010), and like the *Entamoeba* ATP/ADP transporter (Chan et al., 2005) was also shown to localize to yeast mitochondria (Dolezal et al., 2010), suggesting both proteins retain functional mitochondrial targeting signals.

Microsporidians appear to have lost all MCF from their genome (Katinka et al., 2001; Cuomo et al., 2012; Heinz et al., 2012) with the exception of a single protein found in an EST library of the microsporidian *Antonospora locustae* (Williams et al., 2008), subsequently characterized as an ADP/ATP carrier (Williams et al., 2008). This transporter, which was not experimentally verified as being in the *Antonospora* mitosome, more closely resembles FAD/NAD/folate transporters but was demonstrated to transport ATP and ADP with similar affinity and not NAD (Williams et al., 2008). Competition assay data also revealed that the transporter was highly specific for these two nucleotides (Williams et al., 2008) while inhibitor studies showed no inhibition by compounds that are usually effective against archetypal AACs—collectively suggesting that ATP/ADP transport by this unusual transporter may be a novel and recently acquired function (Williams et al., 2008).

## Parasite sugar transporters

Hexose sugars are a crucial source of energy for many parasites. Indeed, parasites such as *P. falciparum* do not appear to maintain energy stores (Sherman, 1979) and glucose deprivation results in a rapid drop in ATP levels in this parasite (Saliba and Kirk, 1999), which is dependent on host glucose during its replication in red blood cells (Krishna et al., 2000; Woodrow et al., 2000). While many parasites have lost genes for a fully functional electron transport chain, they have generally retained the ability to use glucose as a rapid source of ATP through glycolysis.

Several parasitic protists including species of *Leishmania, Plasmodium*, and *Trypanosoma* (Landfear, 2010; Pereira and Silber, 2012) possess hexose transporters that are related in sequence to the mammalian GLUT1, an equilibrative glucose transporter (that facilitates transport down concentration gradients) and is a member of the major facilitator superfamily (MFS). Early work using heterologous expression of the *P. falciparum* transporter PfHT1 in *Xenopus* oocytes, showed it was a sodium-independent glucose transporter but unlike GLUT1 it also transported fructose (Woodrow et al., 2000). It is unclear what physiological role fructose uptake has during parasite infection, as fructose concentration in mammalian blood is generally low, but it may be important during parasite infection of the insect host (Woodrow et al., 2000). Recently, the substrate range of PfHT1 (and PbHT1 from *P. berghei*) has been extended to include other hexose sugars including mannose, fructose, glucose, and galactose (Blume et al., 2011)—with glucose and mannose the preferred substrates (Blume et al., 2011). PfHT1 (and PbHT1) localizes to the parasite plasma membrane (Woodrow et al., 1999; Blume et al., 2011) and is expressed in all life cycle stages of the parasite (Blume et al., 2011), thus highlighting its importance. Indeed, the essential nature of *Plasmodium* hexose transporters has been shown both genetically (Slavic et al., 2010) and chemically using a glucose analog (compound 3361), which inhibits the transport by PfHT1 and kills the parasites in culture and during infection (Joet et al., 2003, 2004). A remarkable finding with PfHT1 is that a single amino acid substitution (Q196N) renders this transporter unable to transport fructose but preserves glucose transport at wild type levels (Woodrow et al., 2000), indicating that this residue is selectively important for substrate specificity. The genome sequence of *P. falciparum* has at least two other putative sugar transporters (PFI0785c and PFI0955w) that are more divergent than PfHT1 from known glucose transporters (Martin et al., 2005). Analysis of the transcripts of these three transporters revealed that they are all expressed during the intra-erythrocyte life cycle, with PfHT1 expressed very early during infection, while the other two are expressed much later (Martin et al., 2005).

Hexose transporters that have been characterized in other parasites include THT1 and THT2 in *T. brucei*, that are related to GLUT-1. Both transporters are highly similar (82% identical) and as with the *Plasmodium* hexose transporters, transport fructose and glucose (Barrett et al., 1998)—with glucose and fructose analogs killing the parasite (Azema et al., 2004). Both are expressed at different stages of the parasite lifecycle, with THT1 expressed in the blood stage while THT2 is expressed constitutively (Krishna et al., 2000). Characterization of *Leishmania* hexose transporters has mainly been done for *L. mexicana*, which has three GLUT1-like glucose transporters—a low affinity isoform termed LmGT1, and two high affinity isoforms termed LmGT2 and LmGT3 (Burchmore et al., 2003; Landfear, 2010). Although these proteins share high sequence similarity, they have different subcellular locations: LmGT2/3 are found in the plasma membrane of the cell body and LmGT1 is located in the membrane of the flagellum (Burchmore et al., 2003). Triple knockout of these three genes in *Leishmania mexicana* rendered the parasite defective in glucose transport and unable to replicate in macrophages (Burchmore et al., 2003). Complementation studies have revealed that the cell body-located LmGT2 and LmGT3, but not the flagellar-located LmGT1, could rescue the mutant (Burchmore et al., 2003). Like the *P. falciparum* transporter PfHT1, the substrate ranges of the three *L. mexicana* transporters and the *T. gondii* transporter TgGT1, include glucose, galactose, fructose, and mannose (Joet et al., 2002; Rodriguez-Contreras et al., 2007; Blume et al., 2009)—substrates that are important in many metabolic pathways.

### Ribose—an alternative substrate for leishmania sugar transporters

Transporters for non-hexose monosaccharides are known in bacteria (Iida et al., 1984) and more recently in plants (Klepek et al., 2005) but have not generally been reported for other eukaryotes. The remarkable substrate range of the *Arabidopsis thaliana* transporter AtPMT5, includes hexoses, pentoses, polyols, and inositol (Klepek et al., 2005). As discussed above, the *Leishmania* hexose transporters are all GLUT1-like family homologs that transport hexoses (Burchmore et al., 2003), but recently LmGT2 and LmGT3 were shown to also transport the pentose sugar D-ribose, the first known example of a ribose transporter in protists (Naula et al., 2010). Ribose is an important metabolic precursor and source of carbon, and in the absence of glucose may be an essential nutrient for *Leishmania* (Steiger and Black, 1980). Strikingly, when a single pair of threonine residues in an extracellular loop of LmGT3 was replaced with alanine, as found in LmGT2, the transport of ribose increased dramatically (Naula et al., 2010)—to a level seen with the latter transporter (Naula et al., 2010).

## Parasite amino acid transporters—the AAAP family

Amino acids serve many biological functions including regulation of osmotic stress, precursors in several metabolic pathways, protein biosynthesis, and alternative sources of energy and carbon (Wu, 2009). Metabolic maps (Kanehisa and Goto, 2000) (http://www.genome.jp/kegg/) for different parasites reveal that they have often lost key enzymes needed to make many amino acids *de novo* and therefore must rely on import from the host. Amino acid import is therefore essential for parasitic protists, yet only a few amino acid transporters have been functionally characterized.

The AAAP (amino acid auxin permease) transporter family (Saier, 2000) have 10–12 transmembrane domains and may transport one or several different amino acids (Saier, 2000). Genome sequence analyses show that multiple copies of AAAP transporters are found in different parasite lineages (Figure 2) (Bouvier et al., 2004) but while some species like *T. brucei* have over 80 predicted AAAPs, others such as *P. falciparum* and *E. cuniculi* have just one or two respectively (TransportDB; Figure 2), suggesting fundamental differences in amino acid requirements or salvage mechanisms. The single predicted AAAP in *P. falciparum* (TransportDB; Figure 2) reflects the parasite's ability to internalize and digest haemoglobin from the host (Wunderlich et al., 2012), a valuable source of amino acids. However, haemoglobin is absent in isoleucine and low in methionine, which likely explains why the parasite imports these amino acids (Cobbold et al., 2011), although the transporter(s) responsible has not yet been identified.

Proline uptake by *Leishmania donovani* is mediated by a low affinity but high capacity transporter called LdAAP24, and homologs of this transporter have been found in *Trypanosoma brucei* (TbAAP24) and *T. cruzi* (TcAAP24). When expressed in yeast mutants deficient in transport of specific amino acids, all three of these parasite transporters rescued the growth defect of the proline-deficient strain while competition and uptake assays with radio-labeled proline revealed selective uptake of proline and a weaker preference for alanine (Inbar et al., 2013). LdAAP24 was localized to the parasite plasma membrane (Inbar et al., 2013) and has been shown to play an important role in the parasite's ability to respond to hypertonic stress (Inbar et al., 2013).

*L. donovani* and *T. cruzi* also have dedicated plasma membrane transporters for the selective uptake of arginine (LdAAP3 and TcAAP411) and lysine (LdAAP7 and TcAAP7) (Shaked-Mishan et al., 2006; Inbar et al., 2012). The reason for separating lysine and arginine transport in these parasites is unclear (mammalian transporters typically transport both amino acids) but it may be related to the fluctuating metabolic demands of these parasites during their life cycle, which may require differential regulation of lysine and arginine salvage.

## Aquaporins—more than just water transporters

Aquaporins (AQP) are a widely distributed family of transporters that belong to the major intrinsic protein (MIP) family that facilitate the bi-directional movement of water in the direction of a concentration gradient (Beitz, 2005). However, in parasitic protists, AQPs exhibit increased functionality with a much broader substrate range compared to classical AQPs such as human AQP1 (Verkman, 2011). Several parasite AQPs have been identified (Figure 2), including the *P. falciparum* PfAQP, *T. gondii* TgAQP, *E. cuniculi* EcAQP, and several AQPs in *Leishmania* species (given below). Interestingly, no AQP gene has been identified in the genomes of *Cryptosporidium parvum* or *Entamoeba histolytica*.

Functional studies on several parasite AQPs reveal that in addition to water, they can transport glycerol, an important precursor of phospholipids and abundant in host serum. *P. falciparum* has a single AQP (PfAQP) (Hansen et al., 2002) that is essential (Promeneur et al., 2007) and expressed throughout the parasite's life cycle (Promeneur et al., 2007). PfAQP is strikingly similar in sequence to the *Escherichia coli* glycerol transporter GlpF (Hansen et al., 2002) but functionally they differ as PfAQP transports both glycerol and water (Hansen et al., 2002), minimizing the need for separate parasite transporters. By contrast, *E. coli* uses GlpF to transport glycerol and another protein (AQPZ) to transport water. Subsequent work has revealed that PfAQP mediates the transport of water, glycerol, sugar alcohols, urea, and ammonia (Hansen et al., 2002; Zeuthen et al., 2006)—suggesting that this transporter may function to remove these latter products from the parasite cytosol.

Transport of glycerol, urea and ammonia has been observed with AQPs from several other parasitic protists (Pavlovic-Djuranovic et al., 2003; Zeuthen et al., 2006; Mandal et al., 2012). *L. donovani* has five divergent AQPs (Biyani et al., 2011), that include a single bacterial-like transporter (LdAQP) and four plant-like AQPs (Biyani et al., 2011). GFP tagging of the *Leishmania* AQPs, revealed several distinct subcellular localizations (Biyani et al., 2011), including the nuclear membrane, the flagella and flagellar pocket (Biyani et al., 2011), although it is possible that the large GFP fusion could have perturbed normal localization. Similarly, an AQP from *L. major* (LmjAQP1), which transports water, glycerol, and several other solutes (Figarella et al., 2007), was found in the flagellar membrane (Mandal et al., 2012) but remarkably was relocated to the entire surface of the parasite when phosphorylated by LmjMPK2, a parasite MAP Kinase (Mandal et al., 2012). This work elegantly shows that parasites are able to rapidly modulate their surface transporters, enabling them to respond to changing metabolic demands during their life cycle. Interestingly, the single AQP in the microsporidian *Encephalitozoon cuniculi* (Ghosh et al., 2006) suggests that not all parasite AQPs have a broad substrate range, as it was found to transport water but not urea or glycerol in *Xenopus* oocytes, similar to classical AQP (Ghosh et al., 2006).

## The ATP-binding cassette (ABC) superfamily

ABC transporters constitute one of the largest protein families in both eukaryotes and prokaryotes (for review see Davidson et al., 2008). They are comprised of two homologous halves, each with an ATP-binding domain located in the cell cytoplasm and a hydrophobic transmembrane domain, while ABC half-transporters contain one of each (Rees et al., 2009). Hydrolysis of ATP provides the energy for the unidirectional transport of a broad spectrum of substrates including sugars, vitamins, proteins, peptides, ions, toxins, and drugs (Rees et al., 2009). Unlike bacterial ABC transporters which are importers and exporters (Rees et al., 2009), eukaryotic ABC transporters are characterized almost exclusively as substrate *exporters*, required for the elimination of a variety of anti-parasitic compounds and conferring multi-drug resistance to the parasite (reviewed elsewhere Klokouzas et al., 2003; Sauvage et al., 2009). At present, there are no known examples of ABC transporters that can function in both directions (Rees et al., 2009).

Although ABC importers were believed, until recently, to be restricted to prokaryotes (Davidson et al., 2008; Locher, 2009; Rees et al., 2009), a few examples have been found in plants (Lee et al., 2008; Kang et al., 2011), yeast (Wilcox et al., 2002), mammals (Quazi et al., 2012), and a recent finding in the apicomplexan parasite *Toxoplasma gondii* (Ehrenman et al., 2010). Six ABCG half transporters were found in *T. gondii*, five of which facilitated the export of cholesterol and phosphatidylserine when expressed in mammalian COS cells. However, one transporter named TgABC_107_ caused a five-fold accumulation of cholesterol, suggesting this protein is an ABC importer (Ehrenman et al., 2010). As TgABC_107_ was localized to the parasite plasma membrane and parasitophorous vacuole (Ehrenman et al., 2010), and its expression was regulated by exogenous addition of sterol to the culture medium, this suggests this transporter may play an important role in cholesterol import from the host cell (Ehrenman et al., 2010).

Genome sequence analysis has revealed that ABC transporters are widely distributed in parasitic protists and comprise one of the largest transporter families (Figure 2) (Kay et al., 2012), ranging from less than fifteen (in microsporidia) to almost a hundred proteins in *T. vaginalis* (Figure 2). Some ABC transporters have been localized to the plasma membrane of parasites including *C. parvum* CpABC (Zapata et al., 2002), *Leishmania infantum* LiABCG4 (Castanys-Munoz et al., 2007), and *P. falciparum* PfMRP1, PfMRP2, and PfMDR5 (Kavishe et al., 2009). Most parasite ABC transporters have been linked to drug resistance and for this reason are well studied, but their physiological roles are poorly understood. Exceptions include LiABCG4 and LiBCG6 that are required for the outward export of the lipid phosphatidylcholine (Castanys-Munoz et al., 2007; Campos-Salinas et al., 2013), which may function to establish the asymmetry between inner and outer leaflets of the plasma membrane (Castanys-Munoz et al., 2007). By contrast, LiABCG5 participates in the salvage of the haem released after the breakdown of internalized haemoglobin (Carvalho et al., 2009; Campos-Salinas et al., 2011). Hence, while ABC transporters are best characterized as exporters in eukaryotes and parasitic protists, their role in the importation and salvage of nutrients may be an important component of the parasitic lifestyle.

## Other transporters

Although the main body of research on parasite transporters has focused on those that transport hexoses and nucleosides, the transporter repertoire in parasites suggest many other transporters mediate nutrient salvage and these are likely to be essential to parasite survival. However, only a few of these transporters have been functionally characterized to date.

### Iron and haem transport

Acquisition of iron is essential for all organisms including parasites, which cannot grow without it (Wilson and Britigan, 1998) and restricting access to iron is a key defense strategy used by macrophages against intracellular infection (Weinberg, 1999). The soluble form of iron is ferrous (Fe^2+^) iron, which is toxic and therefore in short supply within the host cell cytosol. Sequence similarity searches of the *Leishmania amazonensis* genome revealed two genes designated *Leishmania* Iron Transporter 1 and 2 (LIT1 and LIT2) with homology to *Arabidopsis thaliana* IRT1, an Fe^2+^ transporter from the ZIP (ZRT/IRT-like Protein) family of zinc and iron transporters (Guerinot, 2000; Huynh et al., 2006). LIT1 localizes to the parasite plasma membrane and was found to transport Fe^2+^ and promote intracellular survival in macrophages (Huynh et al., 2006). Deletion of LIT1 from *L. amazonensis* revealed it was essential during parasite infection of macrophages and mice but not essential in axenic culture (Huynh et al., 2006), suggesting that other mechanisms for iron acquisition exist in this parasite (Huynh et al., 2006). Interestingly, the ZIP-like transporters family are present in several other parasite genomes (see Figure 2) and have been expanded in some parasites such as the microsporidia (Nakjang et al., 2013).

*Leishmania* can also acquire haem, an iron-containing prosthetic group, either directly via the haem transporter LHR1 (Huynh et al., 2012) or indirectly by endocytosis of haemoglobin (Campos-Salinas et al., 2011). LHR1 was identified in *L. amazonensis* by sequence similarity to the *C. elegans* haem transporter HRG-4, and found to localize to the parasite plasma membrane and lysosome (Huynh et al., 2012). LHR-1 directly imports radio-labeled haem when expressed in yeast (Huynh et al., 2012) and gene deletion demonstrated the importance of LHR1 in haem uptake, parasite replication, and virulence (Miguel et al., 2013). LHR1 orthologs are present in other parasite genomes (Huynh et al., 2012), supporting previous findings that such parasites can take up haem directly from their environment (Cupello et al., 2011).

### Inorganic phosphate transport

Inorganic phosphate (P_i_) is essential in cell metabolism, needed for the synthesis of nucleic acids and for numerous metabolic pathways. The genome of *P. falciparum* contains an inorganic phosphate transporter called PfPiT (Saliba et al., 2006)—a member of the PiT family of H^+^- or Na^+^-coupled P_i_ transporters which are found in prokaryotes and eukaryotes (Saliba et al., 2006). PfPiT was localized to the parasite plasma membrane and found to drive uptake of inorganic phosphate in a Na^+^-dependent manner (Saliba et al., 2006). The sodium dependency of PfPiT and other transporters in *P. falciparum* may explain why the parasite induces a large influx of sodium into infected erythrocytes—the sodium gradient acting as the driving force for the influx of solutes into the parasite (Saliba et al., 2006). The distribution of PiT family members is patchy among investigated parasitic protists (Figure 2) (Saliba et al., 2006), suggesting that alternatives routes of P_i_ uptake exist.

### Folate transport

Folic acid and its derivatives are cofactors for the biosynthesis of purines and amino acids. Several types of folate transporters have been described, and one family, the folate biopterin transporter (FBT) family was initially identified in *Leishmania*. Indeed, *Leishmania* are dependent on an external source of folate for survival (Vickers and Beverley, 2011). The FBT family are distantly related to the MFS, and have only been characterized in protozoa (see below), *A. thaliana* (Klaus et al., 2005) and cyanobacteria (Klaus et al., 2005). There are at least 14 members of this family in *Leishmania* (termed FT1-14) and some have been functionally characterized. Deletion of FT1 in *L. infantum* revealed it was the main folate transporter (Richard et al., 2004) and GFP tagging showed localization to the parasite plasma membrane (Richard et al., 2004) while FT5 has been shown to transport folate with extremely high affinity (Richard et al., 2002). FT1 and FT5 genes are reportedly missing in *Leishmania* mutants that that are resistant to the anti-folate drug methotrexate, suggesting these transporters mediate import of this drug in sensitive strains (Richard et al., 2002, 2004). Two folate transporters, called PfFT1 and PfFT2, that belong to the FBT family were identified in the genome of *P. falciparum* (Salcedo-Sora et al., 2011). This parasite is able to synthesize folic acid *de novo* but is also capable of folic acid uptake *in vitro* (Tan-ariya et al., 1987). Both PfPT1 and PfPT2 localized to the parasite plasma membrane and transported folates when expressed in the *Xenopus* oocyte system (Salcedo-Sora et al., 2011).

### Pantothenic acid transport

Pantothenic acid is a water-soluble vitamin and an essential precursor of CoA, the universal carrier of activated acyl groups. *P. falciparum* cannot synthesize pantothenic acid *de novo* (Saliba et al., 1998; Augagneur et al., 2013) and it must be acquired from its host. Screening a *Plasmodium* cDNA library in yeast identified a gene that complemented yeast mutants defective in pantothenic acid transport (Augagneur et al., 2013). This gene encoded a transporter, named PfPAT that was subsequently found to localize to the plasma membrane (Augagneur et al., 2013). PfPAT is an MFS family member and orthologs of this protein are found throughout the *Plasmodium* genus (Augagneur et al., 2013) and in other parasitic species including *Toxoplasma gondii* and *Cryptosporidium parvum*—underlining the potential importance of pantothenic acid uptake for these parasites (Augagneur et al., 2013).

### Choline transport

Choline is a precursor of phospholipids and is known to be imported by several parasites including species of *Leishmania* (Zufferey and Mamoun, 2002), *Plasmodium* (Kirk et al., 1991) and *Trypanosoma*—suggesting they possess a choline transporter. Choline influx in *P. falciparum* is an electrogenic process, requiring a membrane potential at the parasite plasma membrane (Lehane et al., 2004). While no parasite choline transporter(s) has been identified in these species, specific choline analogs were found to inhibit transport and kill the parasites (Zufferey and Mamoun, 2002; Biagini et al., 2004; Macedo et al., 2013)—strongly supporting the view that parasite choline transporters exist and are essential for survival. Although proteins have been identified that are choline-transporter-like (CTL) in the genome of parasites including microsporidia and *E. histolytica* (Figure 2), putative CTL transporters in *Trypanosoma brucei*, were not reportedly involved in choline uptake as demonstrated by knock down with RNAi (Macedo et al., 2013).

## Conclusions

The notion of stealing nutrients from a host cell is an intriguing area of biology that is still relatively poorly understood. Nutrient transporters constitute a fascinating group of membrane proteins and the few parasite transporters that have been characterized experimentally are often essential for the parasite (Table 1) and therefore represent excellent potential therapeutic targets. Parasite genomes contain a variety of different transporter types and these are often in high copy number (Figure 2) consistent with a general importance for parasite biology. Importantly, parasite genomes also contain a large number of lineage-specific genes of unknown function that have sequence features characteristic of transport proteins. For example, the gene-sparse genome of the microsporidian *Trachipleistophora hominis* contains 52 genes that encode transmembrane domains typical of transporter proteins, but that share no significant similarity to known proteins (Heinz et al., 2012). This theme is repeated among other parasitic protists, and it is likely that these proteins play important roles in specific host-parasite processes, many of which are still to be discovered.

### Conflict of interest statement

The authors declare that the research was conducted in the absence of any commercial or financial relationships that could be construed as a potential conflict of interest.
